# Consumption and drinking frequency of alcoholic beverage among women in Ghana: a cross-sectional study

**DOI:** 10.1186/s12889-015-1651-3

**Published:** 2015-04-01

**Authors:** Anthony Mwinilanaa Tampah-Naah, Samuel Twumasi Amoah

**Affiliations:** Department of Environment and Resource Studies, University for Development Studies, Wa Campus, Wa, Ghana

**Keywords:** Consumption, Drinking frequency, Alcoholic beverage, Women, Ghana

## Abstract

**Background:**

The consumption and drinking frequency of alcoholic beverage are identified with various individual factors. The aim of this study was to identify background characteristics of women associated with the consumption and drinking frequency of alcoholic beverage.

**Methods:**

Data was extracted from the 2008 Ghana Demographic and Health Survey. The data consisted of women’s (aged 15–49 years) background characteristics and their reported history of consumption and drinking frequency of alcoholic beverage. A weighted sample size of 4916 women was used in the present study. Binary logistic regression and multinomial logistic regression were applied to examine the independent association between the covariates and the consumption and drinking frequency of alcoholic beverage respectively.

**Results:**

Out of the 4916 women that were included in the study, 17.5% consumed alcoholic beverage in the past week. Factors that were found to be associated with women who consumed alcoholic beverage in a binary logistic regression model were age (15–19 years up to 45–49 years), region (Central, Greater Accra, Volta, Ashanti, Northern, Upper East, and Upper West), ethnicity (Ga or Dangme, Mole-Dagbani, Grussi, Gruma or Mande), wealth quintile (middle), and employment status [past 12 months] (those employed). In the multinomial logistic regression model, drinking frequency of alcoholic beverage was associated with women in the Central (none), Greater Accra Region (none and 4 or more times), Eastern (none and 2–3 times), Brong Ahafo (none), Upper East (none), those who attained primary education (4 or more times), Ga/Dangme ethnic group (none), those of middle wealth quintile (none), and those employed (4 or more times).

**Conclusions:**

In the country, about 2 in 10 women consume alcoholic beverage and the drinking frequency of alcoholic beverage varied among women in the country. Hence, the maintenance of moderate alcoholic beverage consumption among women, where applicable, should be encouraged.

## Background

Alcoholic beverage consumption is a global phenomenon which is considered as a public health priority worldwide [1,2]. According to Lim et al. [3], alcohol consumption moved from being the eighth highest ranked risk factor in 1990 to the fifth highest ranked risk factor in 2010. The mere consumption of alcoholic beverage by any person may not predispose them to health or social consequences. It is rather the quantity of alcoholic beverage consumed and the drinking frequency that, importantly, can predict the likely health effects or other socially related consequences they encounter [4-6].

One segment of the alcohol consumption debate that has attracted enormous attention in most literature has been the recommended daily alcohol consumption [7,8]. In the alcohol consumption discourse, guidelines are unclear as to the preferred quantity to consume. In one epidemiological research, heavy or at risk drinking for women is defined as more than three drinks a day and for men as more than four drinks in a day [9,10]. Also, the Dietary Guidelines for Americans describe moderate alcohol consumption as drinking up to one drink per day for women and up to two drinks for men [11]. In South Africa, women are advised not to drink more than two ‘standard drinks’ a day and men are not to drink more than three ‘standard drinks’ a day. According to this guideline, ‘standard drink’ means 1 can of beer (340 ml), 1 tot of spirits, 1 glass of white or dry red wine (125 ml) or 1 small glass of sherry (60 ml) [12]. Although a defined level of alcohol consumption is unclear in the literature, alcohol consumption may be influenced by a person’s age, gender, body weight, height, economic status and health status.

The consumption of alcoholic beverage is associated with certain health benefits and risks. Numerous studies have revealed that consuming alcoholic beverage moderately have some health benefits to the consumer; such as sharp reduction in heart disease risk [9], low mortality [13,14] and reduced risk of stroke by about half [15]. The benefits of moderate alcoholic beverage consumption to the heart and others have, therefore, been accepted by many researchers [16,17]. Consuming alcoholic beverage moderately is, hence, linked with better health status and longevity than either abstaining or abusing it [18]. However, the consumption of alcohol moderately, per its general definition, may not be beneficial to everyone; it is advisable to personally and medically examine oneself to determine how moderately to consume alcoholic beverage. On the other hand, heavy consumption of alcoholic beverage can be a prelude to diseases such as cardiovascular diseases [19], indigestion and gastrointestinal ulcers [20], obesity, metabolic syndrome and glucose intolerance [21-23]; reproductive health problems [24] and chronic kidney disease, induced hypertension and liver diseases [25-28].

Globally, it is estimated that 55% of women have never consumed alcoholic beverage [29]. Despite the high abstinence rates of alcoholic beverage consumption, there is a growing concern that there are high risk patterns of drinking among women who have consumed alcoholic beverage in the past year in many low-and middle-income countries [30]. In Ghana, among current drinkers, about 4% of women were heavy alcoholic beverage consumers [31].

Unfortunately, there exist only a draft national alcohol policy intended to be passed by government to guide the consumption, importation, manufacturing, and marketing of alcoholic beverages [32]. This has culminated in paucity and inconsistent information on the consumption of alcoholic beverage in the past decades [33]. Consequently, the control of alcoholic beverage sales and advertisements in the media remains a major challenge in the country [34]. With the absence of a national alcohol policy, the country has continuously witnessed the proliferation of alcoholic beverages with regular advertisements on the media especially televisions, radio stations and on wide bill boards [35], with women playing major roles in most of these alcoholic beverage advertisements [36,37].

The consumption of either foreign alcoholic beverages (such as wines and whiskeys) or locally produced alcoholic beverages such as *pito*, liqueurs (Alomo Bitters, Kasapreko, Striker, Pusher or Ginseng) and gin (Akpeteshie) among women can lead to undesirable outcomes such as unplanned pregnancy [38,39]. Agoabasa [40] noted that women resort to the consumption of alcoholic beverage as a way to cope with stress and anxiety in society. Despite these persistent claims, to the best of our knowledge, there exist a dearth of research in Ghana which presents empirical analysis of the characteristics that bears out the alcoholic beverage consumption and drinking frequency of women. Based on this literature gab, the present study aimed to analyze the characteristics that underpin alcoholic beverage consumption and drinking frequency among women in Ghana; using data from the 2008 Ghana Demographic and Health Survey (GDHS) [33].

## Methods

### Study area

Ghana is located in West Africa and has a total land area of 238, 537 square kilometers [33]. The country’s population is about 24, 658, 823 people. This consists of 12, 633, 978 (51.2%) females and 12, 024, 845 males (48.8%); translating to a sex ratio of 95 males to 100 females [41].

### Study design and sample

The study employed a cross-sectional approach using data set from the 2008 Ghana Demographic and Health Survey (GDHS). The individual (women) data set was specifically used for the data analysis. The GDHSs are national-level population and health surveys conducted in Ghana as part of the global Demographic and Health Survey programme. These surveys are designed to provide information to monitor the population and health situation in Ghana [33].

The 2008 GDHS used a two-stage sample based on the 2000 Population and Housing Census to produce separate estimates for key indicators for each of the ten regions in Ghana. Each household selected for the 2008 GDHS was eligible for interview with a household questionnaire. On the part of women, the survey involved a total of 4,916 women aged 15–49 years and the response rate to the survey questionnaire yielded a rate of 97 percent.

All women (4,916) who took part in the survey were considered for analysis in the present study. Sample size was weighted using the relevant weight (w = v005) as recommended by MeasureDHS. To validate the data, frequencies and percentages of the various characteristics of women were recalculated. And the results were compared to the figures in the 2008 GDHS report for consistency. The usage of this data was approved by MeasureDHS (Demographic and Health Survey) on June 29, 2011through their website http://www.measuredhs.com.

### Dependent variables

The present study considered two dependent variables: Alcoholic beverage consumption and drinking frequency of alcoholic beverage. The first dependent variable, alcoholic beverage consumption, was operationalized as whether a woman (aged 15–49 years old) has consumed, at least one drink of any type of alcoholic beverage in the past week preceding the survey. The term “drink” in the definition can be a bottle of beer/stout, a glass of wine, a shot of liquor/a mixed drink with liquor, or a calabash of *pito*/palm wine. This was guided by the question (as captured in the 2008 GDHS questionnaire), “Do you consume alcoholic beverage?” The expected response to this question was either “Yes” or “No”; these were adopted by the present study.

The second dependent variable, drinking frequency of alcoholic beverage, was defined by the present study as “the frequency (once, 2–3 times, 4 times or more, or not at all) of consuming alcoholic beverage within the past week preceding the survey by a woman of the age cohort 15–49 years old”. The words “drinking” and “consuming” have been used interchangeably in the present study. This definition was informed by a follow up question to the alcoholic beverage consumption; thus the first dependent variable. Those women who responded in affirmative to the alcohol consumption question were further asked “How frequently they had consumed alcohol during the past week: …” (GSS 2009, p.60).

### Independent variables

The independent variables (background characteristics of women) that were used include: age, marital status, residence, region, education, ethnicity, wealth, and employment. Some of the variables were recoded while others were adopted as reported in the 2008 GDHS:

- Age of women (15–49 years old) were coded as: 15–19, 20–24, 25–29, 30–34, 35–39, 40–44, and 45–49.
- Marital status of women was recoded into three groups: Never married, married/cohabiting and divorced/separated/widowed.
- Residence was categorized as urban and rural as captured in the 2008 GDHS.
- Region, all the ten regions in the country were considered. These are Western, Central, Greater Accra, Volta, Eastern, Ashanti, Brong Ahafo, Northern, Upper East and Upper West.
- Education was reported in terms of a woman’s educational level. This variable was recoded as no education, primary and secondary/higher.
- Ethnicity, categories were recoded into five groups: Akan, Ga/Dangme, Ewe/Guan, Mole-Dagbani and Grussi/Gruma/Mande/others.
- Wealth quintile coding was adopted as captured in the 2008 GDHS report: Poor, middle and rich.
- Employment (past 12 months) was captured as not employed and employed.

Independent variables’ categories that had smaller number of frequencies were collapsed or regrouped with similar categories to form larger categories. For instance, marital status, education, ethnicity and wealth quintile categories were regrouped.

### Data analysis

Descriptive statistics were used to present the background characteristics of the women. Also, chi-square test of independence was performed to test for individual independent association (*p* < 0.05) between the independent and dependent variables, and proportions were presented in terms of the women who consumed alcohol. With the drinking frequency of alcohol, summaries were presented in frequencies and proportions.

Further analyses were applied in this study: Binary logistic regression and multinomial logistic regression. All background variables were considered for the binary logistic regression since all were statistically significant (p < 0.05). Only the variable age was not considered for the multinomial logistic regression.

The binary logistic regression was applied to generate a model for women who consumed alcoholic beverage in the past week. This form of analysis was applied since the dependent variable had dichotomous responses; with responses coded as “Yes” = *1* (consume alcoholic beverage) and “No” = *0* (do not consume alcoholic beverage). Initially, unadjusted binary logistic regression model was generated and later adjusted model was built by controlling for all statistical significant (p < 0.05) characteristics.

Similarly, all statistical significant (p < 0.05) characteristics with the drinking frequency of alcohol (none, once, 2–3 times, and 4 times or more) were primarily used to build unadjusted models. Subsequently, adjusted models were generated by controlling for all significant (p < 0.05) independent variables. Women that consumed alcoholic beverage once in the past week before the survey were used as the reference group because they reported most of consuming alcoholic beverage.

Both results of the binary logistic regression and multinomial logistic models were presented as odds ratio with their corresponding 95% confidence intervals (CI). Data processing and statistical analysis were done using STATA version 11.

## Results

Table 1 shows distribution of background characteristics of women that were considered in the study. The study revealed that the pass week alcoholic beverage consumption in the country was 17.5%. From the distribution, there were proportional variations in the consumption of alcoholic beverage among the independent variables (Table 2).

**Table 1:** **Background characteristics of women (N = 4916)**

**Characteristics**	**n**	**%**
**Age**		
15-19	1025	20.8
20-24	878	17.9
25-29	832	16.9
30-34	644	13.1
35-39	638	13.0
40-44	470	9.6
45-49	429	8.7
Marital status*		
Never married	1593	32.4
Married/Cohabiting	2876	58.5
Divorced/Separated/Widowed	446	9.1
**Residence**		
Urban	2383	48.5
Rural	2533	51.5
**Region**		
Western	447	9.1
Central	424	8.6
Greater Accra	853	17.3
Volta	431	8.8
Eastern	483	9.8
Ashanti	1011	20.6
Brong Ahafo	425	8.7
Northern	467	9.5
Upper East	253	5.2
Upper West	122	2.5
Education*		
No education	1042	21.2
Primary	988	20.1
Secondary/Higher	2692	58.7
Ethnicity*		
Akan	2493	50.7
Ga/Dangme	343	7.0
Ewe/Guan	755	15.3
Mole-Dagbani	795	16.2
Grussi/Gruma/Mande	528	10.8
**Wealth**		
Poor	1683	34.2
Middle	979	19.9
Rich	2254	45.8
**Employment (past 12 months)**		
Not employed	1240	25.2
Employed	3676	74.8

n: frequency of variable, %: percentage of frequency, *N < 4916 due to missing responses.

**Table 2:** **Proportions and predictors of alcoholic beverage consumption among women**

**Characteristics**	**Alcohol consumption**		**p-value**	**Age adjusted**		**Multivariate**	
				**COR**	**95% CI**	**AOR**	**95% CI**
	**n = 861**	**% = 17.5**					
**Age**			0.000				
15-19	68	6.6		1		1	
20-24	146	16.7		2.40***	1.81, 3.17	2.40***	1.73, 3.32
25-29	137	16.5		2.63***	1.98, 3.48	2.57***	1.78, 3.72
30-34	133	20.7		3.11***	2.33, 4.16	2.99***	2.02, 4.42
35-39	162	25.4		4.10***	3.09, 5.43	3.82***	2.59, 5.65
40-44	104	22.2		3.51***	2.59, 4.75	2.98***	1.96, 4.52
45-49	111	26.0		4.76***	3.51, 6.43	4.42***	2.91, 6.70
**Marital status**			0.000				
Never married	175	11.0		1		1	
Married/Cohabiting	569	19.8		2.04***	1.71, 2.43	0.87	0.66, 1.13
Divorced/Separated/Widowed	117	26.3		2.85***	2.19, 3.70	1.34	0.94, 1.70
**Residence**			0.000				
Urban	404	17.0		1		1	
Rural	457	18.1		1.40***	1.21, 1.62	1.23	0.98, 1.55
**Region**							
Western	48	10.7		1		1	
Central	70	16.5		2.04**	1.33, 3.14	2.16**	1.39, 3.37
Greater Accra	223	26.2		3.30***	2.29, 4.75	3.77***	2.49, 5.69
Volta	105	24.3		3.17***	2.15, 4.68	2.91***	1.86, 4.54
Eastern	69	14.2		1.65*	1.11, 2.49	1.48	0.97, 2.27
Ashanti	131	13.0		1.43	0.98, 2.11	1.69*	1.13, 2.52
Brong Ahafo	37	8.6		0.95	0.59, 1.52	1.06	0.65, 1.71
Northern	71	15.2		2.25***	1.52, 3.33	3.45***	2.21, 5.41
Upper East	63	25.0		3.21***	2.15, 4.78	5.62***	3.47, 9.12
Upper West	46	37.3		6.28***	4.32, 9.11	11.98***	7.60, 18.90
**Education**			0.000				
No education	222	21.3		1		1	
Primary	178	18.0		0.67***	0.55, 0.82	0.95	0.76, 1.8
Secondary/Higher	461	16.0		0.53***	0.45, 0.62	0.85	0.69, 1.06
**Ethnicity**			0.000				
Akan	359	14.4		1		1	
Ga/Dangme	110	32.0		2.51***	1.91, 3.29	1.66**	1.21, 2.28
Ewe/Guan	182	24.1		1.86***	1.51, 2.28	1.20	0.91, 1.59
Mole-Dagbani	120	15.1		1.73***	1.43, 2.08	0.47***	0.34, 0.67
Grussi/Gruma/Mande	89	16.9		1.75***	1.40, 2.18	0.65**	0.48, 0.87
**Wealth**			0.000				
Poor	323	19.2		1		1	
Middle	133	13.6		0.51***	0.41, 0.64	0.64***	0.50, 0.81
Rich	405	18.0		0.69***	0.59, 0.80	0.87	0.67, 1.13
**Employment (past 12 months)**			0.000				
Not employed	133	10.7		1		1	
Employed	728	19.8		2.16***	1.78	1.46**	1.16, 1.83

n: women who consume alcohol, %: proportion of women who consume alcohol, COR: Crude odds ratio, AOR: Adjusted odds ratio, CI: Confidence Interval, *p < 0.05, **p < 0.01, ***p < 0.001

All the independent variables were considered for generating the binary logistic regression model since all exhibited statistical significant (*p* < 0.05) association with the first dependent variable (alcoholic beverage consumption) (Table 2). Controlling for all variables, categories that were found to be associated with alcoholic beverage consumption among women in the binary logistic regression model were 15–19 years to 45–49 years, Central, Greater Accra, Volta, Ashanti, Northern, Upper East, and Upper West, Ga/Dangme, Mole-Dagbani, Grussi,/Gruma /Mande, middle wealth quintile, and employed women. Women in the 45–49 years category were 4 times more likely to consume alcoholic beverage compared to women in the 15–19 years category (AOR = 4.42; 95% CI: 2.91, 6.70). Women in the Upper West Region were about 12 times more likely to consume alcoholic beverage compared to women in the Western Region (AOR = 11.98; 95% CI: 7.60, 18.90). Women of the Ga or Dangme ethnic group were more likely to consume alcoholic beverage compared to women of the Akan ethnic group (AOR = 1.66; 95% CI: 1.21, 2.28). Likewise, women of the middle wealth quintile were less likely to consume alcoholic beverage compared to those in the poor wealth quintile (AOR = 0.64; 95% CI: 0.50, 0.81). In terms of employment (past 12 months), women who were employed were more likely to consume alcoholic beverage compared to those who were not employed (AOR = 1.46; 95% CI: 1.16, 1.83).

Table 3 provides bivariate associations of the drinking frequency of alcoholic beverage among women. Out of the eight independent variables examined in the bivariate association, only age was not statistical significant (p < 0.05) with drinking frequency of alcoholic beverage.

**Table 3:** **Alcoholic beverage drinking frequency and associated factors among women**

**Characteristic**	**Drinking patterns of drinking alcoholic beverages (past 7 days) (N = 861)**								
	**None**		**Once**		**2-3 times**		**4 times** **or more**		**p-value**
	**n(%)**		**n**	**%**	**n**	**%**	**n**	**%**	
**Age**									0.063
15-19	32	47.6	26	38.4	9	12.9	1	1.1	
20-24	62	42.1	59	40.7	20	13.7	5	3.5	
25-29	40	28.8	54	39.6	34	24.9	9	6.8	
30-34	46	34.6	48	33.5	28	20.7	15	11.6	
35-39	46	28.9	47	29.4	48	30.2	19	11.6	
40-44	29	28.4	42	41.1	25	24.3	6	6.2	
45-49	27	24.2	44	39.2	32	28.4	9	8.2	
**Marital status**									0.000*
Never married	82	46.9	67	38.2	20	11.3	6	3.6	
Married/cohabiting	164	28.9	205	36.3	150	26.5	47	8.2	
Divorced/Separated/Widowed	35	30.1	45	38.6	25	21.7	11	9.5	
**Residence**									0.000*
Urban	178	44.2	147	36.4	61	14.9	18	4.5	
Rural	103	22.7	170	37.5	135	29.7	46	10.1	
**Region**									0.000*
Western	13	27.5	24	51.4	8	17.0	1	4.1	
Central	38	55.3	15	21.9	9	13.5	6	9.3	
Greater Accra	98	44.0	77	34.6	32	14.1	16	7.2	
Volta	12	11.3	57	54.8	29	27.8	6	6.1	
Eastern	20	27.8	16	22.8	25	36.6	7	10.9	
Ashanti	55	42.9	43	33.3	26	20.5	4	3.2	
Brong Ahafo	18	48.9	10	27.4	8	21.2	1	2.4	
Northern	18	25.2	25	35.1	20	28.3	8	11.4	
Upper East	2	2.5	34	55.5	21	34.9	4	7.1	
Upper West	6	13.5	15	32.7	16	35.9	8	17.9	
**Education**									0.000*
No education	43	19.4	86	39.0	71	32.1	21	9.5	
Primary	44	24.9	62	35.6	45	25.4	25	14.2	
Secondary/Higher	194	42.3	168	36.6	79	17.3	18	3.9	
**Ethnicity**									0.000*
Akan	166	46.1	110	30.7	63	17.6	20	5.6	
Ga/Dangme	31	28.6	47	42.6	20	18.5	11	10.4	
Ewe/Guan	49	26.9	75	41.2	46	25.5	12	6.4	
Mole-Dagbani	15	12.7	54	46.1	36	30.7	12	10.6	
Grussi/Gruma/Mande	20	23.0	30	34.3	29	33.1	8	9.7	
**Wealth**									0.000*
Poor	56	17.3	123	38.1	105	32.7	38	11.8	
Middle	56	42.2	40	30.3	25	18.8	12	8.7	
Rich	170	42.1	154	38.3	65	16.1	14	3.6	
**Employment (past 12 months)**									0.037*
Not employed	53	40.7	51	38.6	23	17.4	4	3.2	
Employed	228	31.4	267	36.7	172	23.7	59	8.2	

n: number of women, %: percentage of women who consumed alcohol during the past week preceding the survey. *Statistcally significant (p < 0.05). Note: Some frequencies do not correspond with frequencies in Table 2 due to missing responses.

The multinomial logistic regression models are displayed on Table 4. After controlling for all significant (p < 0.05) independent variables: region, education, ethnicity, wealth quintile and employment exhibited association with drinking frequency of alcoholic beverage. Women in the Central Region were 5 times more likely not to consume alcoholic beverage rather than once compared to women in the Western Region (AOR = 5.34, 95% CI = 7.94, 14.70). Likewise, women in the Eastern Region were found to be 3 times more likely to consume alcoholic beverage 2–3 times rather than once compared to those in the Western Region (AOR = 3.09, 95% CI = 1.03, 9.27). Similarly, women in the Greater Accra Region were 6 times more likely to consume alcoholic beverage 4 or more times rather than once compared to those in Western Region (AOR = 6.19, 95% CI = 1.03, 37.19). On education, women who had primary education were 2 times more likely to consume alcoholic beverage 4 or more times rather than once compared to those with no education (AOR = 2.28, 95% CI = 1.17, 4.42). In terms of ethnicity, women of the Ga or Dangme group were less likely not to consume alcoholic beverage compared to those of the Akan origin (AOR = 0.39, 95% CI = 0.20, 0.77). With wealth quintile, women of middle wealth quintile were 2 times more likely not to consume alcoholic beverage rather than once compared to those in the poor wealth quintile (AOR = 2.26, 95% CI = 1.21, 4.20). On employment, employed women were 3 times more likely to have consumed alcoholic beverage 4 or more times rather than once compared to those who were not employed (AOR = 2.59, 95% CI = 1.01, 6.62).

**Table 4:** **Association of alcohol drinking frequency with background characteristics of women (N = 861)**

**Characteristic**	**Unadjusted**			**Adjusted**		
	**None (vs. Once)**	**2-3 times (vs. Once)**	**≥4 times (vs. Once)**	**None (vs. Once)**	**2-3 times (vs. Once)**	**≥4 times (vs. Once)**
	**OR (95% CI)**	**OR (95% CI)**	**OR (95% CI)**	**AOR (95% CI)**	**AOR (95% CI)**	**AOR (95% CI)**
**Marital status**						
Never married	1	1	1	1	1	1
Married/cohabiting	0.59 (0.41, 0.88)*	1.77 (1.11, 2.81)*	1.79 (0.88, 3.69)	0.85 (0.55, 1.33)	1.63 (0.97, 2.73)	1.61 (0.73, 3.57)
Divorced/Separated/Windowed	0.64 (0.37, 1.12)	1.35 (0.72, 2.55)	1.80 (0.72, 4.51)	0.61 (0.33, 1.12)	1.30 (0.67, 2.54)	1.76 (0.65, 4.73)
**Residence**						
Urban	1	1	1	1	1	1
Rural	0.42 (0.30, 0.58)*	2.05 (1.41, 2.96)*	2.48 (1.38, 4.45)*	0.86 (0.51, 1.45)	1.53 (0.88, 2.66)	1.5 (0.62, 4.02)
**Region**						
Western	1	1	1	1	1	1
Central	5.00 (1.86,13.44)*	1.67 (0.49, 5.61)	4.17 (0.69, 24.94)	5.34(1.94,14.70)*	1.52(0.45,5.22)	3.77 (0.60, 23.71)
Greater Accra	2.57 (1.14, 5.77)*	1.03 (0.40, 2.67)	2.07 (0.43, 10.05)	3.10 (1.27, 7.55)*	1.42(0.49,4.09)	6.19(1.03,37.19)*
Volta	0.49 (0.19, 1.26)	1.45 (0.57, 3.68)	0.91 (0.16, 5.07)	0.55 (0.18, 1.66)	0.92 (0.29, 2.91)	0.92 (0.12, 6.92)
Eastern	2.22 (0.85, 5.83)	3.19 (1.15, 8.91)*	3.89 (0.71, 21.19)	3.36 (1.20, 9.36)*	3.09(1.03,9.27)*	3.89 (0.64, 23.59)
Ashanti	2.23 (0.94, 5.28)	1.57 (0.59, 4.18)	1.14 (0.19, 6.80)	2.10 (0.87, 5.07)	1.60 (0.59, 4.37)	1.21 (0.19, 7.68)
Brong Ahafo	3.09 (1.06, 9.04)*	2.00 (0.58, 6.91)	1.00(0.08,12.39)	4.04(1.28,12.71)*	1.47(0.39,5.57)	0.62 (0.04, 9.08)
Northern	1.21 (0.48, 3.07)	2.50 (0.95, 6.55)	4.67 (0.96, 22.79)	1.96 (0.56, 6.92)	0.98 (0.27, 3.59)	3.21 (0.39, 26.59)
Upper East	0.10 (0.03, 0.39)*	1.29 (0.51, 3.31)	0.93 (0.17, 5.16)	0.18 (0.36, 0.94)*	0.84(0.23,3.11)	0.53 (0.06, 5.11)
Upper West	0.69 (0.28, 1.65)	2.66 (1.09, 6.49)*	4.75(1.04,21.72)*	1.31 (0.38, 4.48)	1.73 (0.49, 6.08)	2.45 (0.31, 19.61)
**Education**						
No education	1	1	1	1	1	1
Primary	1.83 (1.11, 3.03)*	0.92 (0.59, 1.42)	1.63 (0.93, 2.87)	1.17 (0.65, 2.11)	1.09 (0.66, 1.81)	2.28 (1.17, 4.42)*
Secondary/Higher	3.20 (2.16, 4.76)*	0.63 (0.44, 0.92)*	0.44 (0.24, 0.81)*	1.39 (0.79, 2.46)	1.01 (0.60, 1.69)	0.82 (0.36, 1.86)
**Ethnicity**						
Akan	1	1	1	1	1	1
Ga/Dangme	0.44 (0.25, 0.78)*	0.81 (0.42, 1.56)	1.06 (0.43, 2.64)	0.39 (0.20, 0.77)*	0.71 (0.32, 1.57)	0.52 (0.17, 1.55)
Ewe/Guan	0.44 (0.28, 0.69)*	1.17 (0.71, 1.92)	0.72 (0.31, 1.65)	1.02 (0.53, 1.97)	1.35 (0.62, 2.94)	0.59 (0.19, 1.89)
Mole-Dagbani	0.19 (0.11, 0.30)*	1.19 (0.76, 1.88)	1.59 (0.84, 3.02)	0.79 (0.31, 2.03)	1.11 (0.42, 2.92)	1.30 (0.29, 5.67)
Grussi/Gruma/ Mande	0.39 (0.23, 0.68)*	2.31 (1.38, 3.88)*	1.88 (0.88, 4.03)	1.17 (0.44, 3.10)	2.28 (0.87, 6.011)	0.95 (0.22, 4.22)
**Wealth**						
Poor	1	1	1	1	1	1
Middle	3.69 (2.22, 6.14)*	0.84 (0.49, 1.44)	0.84 (0.39, 1.79)	2.26 (1.21, 4.20)*	1.08 (0.58, 1.99)	1.11 (0.46, 2.71)
Rich	3.18 (2.21, 4.57)*	0.52 (0.36, 0.76)*	0.33 (0.18, 0.62)*	1.50 (0.81, 2.78)	0.93 (0.51, 1.70)	0.42 (0.15, 1.17)
**Employment (past 12 months)**						
Not employed	1	1	1	1	1	1
Employed	0.88 (0.58, 1.34)	1.43 (0.89, 2.29)	2.56 (1.06, 6.14)*	1.25(0.77, 2.01)	1.19 (0.71, 2.02)	2.59(1.01, 6.62)*

OR - Crude odds ratio; AOR - Adjusted odds ratio; CI- Confidence Interval; vs - versus *: p < 0.05.

## Discussion

The present study used a nationwide dataset (2008 GDHS) to examine consumption and drinking frequency of alcoholic beverage in the country [33]. The results from the study evidenced that women of various categories in the country do consume alcoholic beverage. Although women in the country do consume alcoholic beverage, caution should be taken when examining the results since these may not indicate any form of risk drinking of alcoholic beverage. Also, it should be noted that the dataset used in the present study was based on a cross-sectional design and the survey’s sample was drawn from women aged 15–49 years. Therefore, the purpose of this present study was to examine associated factors; first with women who consumed alcoholic beverage, and, second, with their drinking frequency of alcoholic beverage in the past week before the survey. It is worth noting that, there were proportional variations in the consumption and the drinking frequency of alcoholic beverage among women. The prevalence rate of alcoholic beverage consumption in the country was 17.5% [33]. For those women who consume alcoholic beverage once or several in a week are likely to have high volume of or low volume as compared to a woman who consumes one drink per week [42]. In a similar way, moderate consumers of alcoholic beverage could also be risk drinkers; considering how quickly they drink alcohol, their age or their health status. The discussion is focused on factors that were statistically significant after controlling for all independent variables both in the binary logistic regression and multinomial logistic regression models on alcohol consumption in the past week.

Regarding alcoholic beverage consumption and age, older women especially those in their thirties and forties had a higher likelihood of consuming alcoholic beverage compared to younger women in their teens and twenties. However, a woman’s age was not a significant factor in determining her drinking frequency of alcoholic beverage. This implies that a woman’s age does not influence the frequency (none, once, 2–3 times, 4 or more times) at which she may consume alcoholic beverage.

It became evident that, women in the Upper West, Upper East, Central, Greater Accra, Volta and Ashanti Regions were found to have higher odds of consuming alcoholic beverage compared to women of the Western Region. Among these regions, women in the Upper West Region were more likely to consume alcoholic beverage. Also, a higher proportion of these women (37%) in the region, compared to other regions, consumed alcoholic beverage 2–3 times. The present study’s finding concurs with a study by Luginaah and Dakubo [38] which suggest that inhabitants of the region increasingly consume alcoholic beverages. The aforementioned study [38] focused on a community’s perception on alcohol use. According to this paper, men consume alcohol mainly for coping responses, for example, increased self-confidence, adult status, and to cope with the various social demands. On the side of women, they seem to consume alcohol for purpose of socializing with their peers. Also, women in the region consume alcoholic beverage, perhaps, due to the insufficient sustainable means of livelihood and gainful employment. On the other hand, the region is noted with *pito* (locally brewed alcoholic beverage), and this too can contribute to why women reported that they consume alcohol. In addition to the *pito*, other alcoholic beverages are readily available to women in the region. Since the 2008 GDHS did not specify the kind of alcoholic beverage consumed by the women, the present study can conclude that some women in the region consume alcoholic beverage; which speaks little of the volume of alcoholic beverage they consume. Hence, it was not enough to also conclude in the present study that women in the Upper West Region frequently consume alcoholic beverage. By controlling for all significant background characteristics, it was revealed that women in the Upper West Region once a while consume alcoholic beverage but rather women in the Eastern Region were more likely to consume alcoholic beverage 2–3 times and those in the Greater Accra Region were more likely to consumed alcoholic beverage 4 or more times. Probably women in these regions are equally engaged in social occasions where alcoholic beverages are at their disposal or they generally have a higher tendency of consuming alcoholic beverage frequently. Longitudinal studies may be needed to better understand differences in the consumption and the drinking pattern (in terms of volume) of alcoholic beverage in the country. Also, more detailed surveys that take into account a variety of aspects of alcohol use, such as context, motivations and expectations, would be useful for improving the understanding of alcohol use in the country.

The present study further revealed that women of the Grussi, Gruma, or Mande, Ga/Dangme, and Mole-Dagbani ethnic groups commonly consumed alcoholic beverage compared to those of the Akan ethnic group. In addition, Grussi, Gruma or Mande women reported more of ever consuming alcoholic beverage. However, higher proportions of women from these ethnic groups consumed alcoholic beverage once in the past week. These findings can be attributed to the fact that these ethnic groups are found in regions that are associated with alcohol consumption; predominately, Grussi, Gruma or Mande in the Upper East Region, Mole-Dagbani in the Northern Region and Ga/Dangme in the Greater Accra Region. It will be worthwhile for community based studies to be conducted to assess the consumption and drinking frequency of alcoholic beverage among various homogenous groups.

Alcoholic beverage consumption has been found to be associated with economic status of an individual [43]. In the present study, women of the middle and rich wealth quintiles were less likely to consume alcoholic beverage compared to those in the poor wealth quintile. The present study’s analysis reflects that most women had attained at least secondary education and this implies that they are likely to be in a higher economic status. Hence, these higher economic status women would be in a better position to make well informed decisions by not initiating the consumption of alcoholic beverage. However, when all significant factors were controlled in the multinomial regression analysis, it further showed that women of the middle wealth quintile were less likely not to have consumed alcoholic beverages. This outcome can be attributed to the educational level and better access to information that tend to inform them to drink less alcoholic beverage.

In line with results of a study by Martinez et al. [31], the present study found that women who were employed reported of consuming alcoholic beverage and were 4 or more times more likely to have consumed alcoholic beverage compared to those who were not employed. Martinez et al. [31] found out that women working for pay had a higher likelihood of being current consumers in Mauritius, Chad and Ghana. Probably, women who are involved in part-time works or who were employed in the past 12 months before the survey had a higher purchasing power for alcoholic beverage compared to those who were not employed. It can also be that this category of women tend to socialize more and in the process are more likely to consume alcoholic beverage.

The findings of this study should be considered in relation to a number of limitations. One limitation is the generalized nature in which alcohol data were collected among the women in the past week. Another is the collapsing of some variable categories that might have affected them been significant or otherwise. The study design (cross-sectional) limits the present study’s ability to draw inferential interconnections between the independent variables and the consumption and drinking frequency of alcoholic beverage in the country. Other limitations associated with the present study are the inherent bias (over reporting or underreporting) in self-report and the limited data collection time frame for the consumption and drinking frequency of alcoholic beverages. Beside, limited related references were used to support or refute the present study’s findings. Also, the study acknowledges the lack of any quantity measure of alcohol use among women. Notwithstanding these limitations, the 2008 GDHS presents reliable alcohol related datasets that can be considered as the benchmark of the consumption and drinking frequency of alcoholic beverage surveys. Based on these limitations, the present study suggests to the Ghana Statistical Service, which is mainly responsible for conducting the GDHS, to include better measures of alcohol use in future surveys. However, the present study’s findings are reflective of women’s consumption and drinking frequency of alcoholic beverage in the country.

## Conclusions

In the country, there was an indication of alcohol consumption among women. Women of all age groups across the regions consume a certain quantity of alcoholic beverage. It can also be concluded that, really, women do consume alcoholic beverage in the country. The study recommends that the Ministry of Health in collaboration with the media should design and rollout programmes on the maintenance of moderate consumption of alcoholic beverage among women. Also, more studies are needed to assess the consequences and reasons of drinking frequency of alcoholic beverage. To better apply interventions, it will be cost-efficient to cover regions or groups that are likely to consume alcoholic beverage. This will facilitate in drawing a comprehensive national alcohol policy in the country.
